# Regulation of 5'-adenosine monophosphate deaminase in the freeze tolerant wood frog, *Rana sylvatica*

**DOI:** 10.1186/1471-2091-9-12

**Published:** 2008-04-22

**Authors:** Christopher A Dieni, Kenneth B Storey

**Affiliations:** 1 Institute of Biochemistry and Department of Chemistry, Carleton University, 1125 Colonel By Drive, Ottawa, Ontario, K1S 5B6, Canada

## Abstract

**Background:**

The wood frog, *Rana sylvatica*, is one of a few vertebrate species that have developed natural freeze tolerance, surviving days or weeks with 65–70% of its total body water frozen in extracellular ice masses. Frozen frogs exhibit no vital signs and their organs must endure multiple stresses, particularly long term anoxia and ischemia. Maintenance of cellular energy supply is critical to viability in the frozen state and in skeletal muscle, AMP deaminase (AMPD) plays a key role in stabilizing cellular energetics. The present study investigated AMPD control in wood frog muscle.

**Results:**

Wood frog AMPD was subject to multiple regulatory controls: binding to subcellular structures, protein phosphorylation, and effects of allosteric effectors, cryoprotectants and temperature. The percentage of bound AMPD activity increased from 20 to 35% with the transition to the frozen state. Bound AMPD showed altered kinetic parameters compared with the free enzyme (*S*_0.5 _AMP was reduced, Hill coefficient fell to ~1.0) and the transition to the frozen state led to a 3-fold increase in *S*_0.5 _AMP of the bound enzyme. AMPD was a target of protein phosphorylation. Bound AMPD from control frogs proved to be a low phosphate form with a low *S*_0.5 _AMP and was phosphorylated in incubations that stimulated PKA, PKC, CaMK, or AMPK. Bound AMPD from frozen frogs was a high phosphate form with a high *S*_0.5 _AMP that was reduced under incubation conditions that stimulated protein phosphatases. Frog muscle AMPD was activated by Mg·ATP and Mg·ADP and inhibited by Mg·GTP, KCl, NaCl and NH_4_Cl. The enzyme product, IMP, uniquely inhibited only the bound (phosphorylated) enzyme from muscle of frozen frogs. Activators and inhibitors differentially affected the free versus bound enzyme. *S*_0.5 _AMP of bound AMPD was also differentially affected by high versus low assay temperature (25 vs 5°C) and by the presence/absence of the natural cryoprotectant (250 mM glucose) that accumulates during freezing.

**Conclusion:**

Maintenance of long term viability under the ischemic conditions in frozen muscle requires attention to the control of cellular energetics. Differential regulatory controls on AMPD by mechanisms including binding to muscle proteins, actions allosteric effectors, glucose and temperature effects and reversible phosphorylation adjust enzyme function for an optimal role in controlling cellular adenylate levels in ischemic frozen muscle. Stable modification of AMPD properties via freeze-responsive phosphorylation may contribute both to AMPD control and to coordinating AMPD function with other enzymes of energy metabolism in cold ischemic muscle.

## Background

Seasonal temperatures below 0°C are a major challenge to viability for terrestrial ectotherms around the world. In many cases, biochemical adaptations supplying cold tolerance are key to winter survival [1,2]. For many species, cold tolerance includes the ability to endure the freezing of body fluids and, among vertebrates, this capacity has been best-studied in a species that hibernates on the forest floor, the wood frog, *Rana sylvatica *(also known as *Lithobates sylvaticus*) [3]. Prominent adaptations that underlie wood frog freeze tolerance include the accumulation and distribution of huge amounts of glucose as a colligative cryoprotectant [4-7], the synthesis of ice-nucleating proteins [8], selective up-regulation of a variety of freeze-responsive genes [9], and metabolic rate depression [10,11].

The freezing of 65–70% of total body water in extracellular and extra-organ spaces halts heart beat and breathing and rapidly leads to conditions of anoxia and ischemia [3,12]. These conditions place energy stress on cells and over time the tissues of frozen frogs build up glycolytic end products (lactate, alanine) and deplete ATP [13]. This reduces the Adenylate Energy Charge (AEC): AEC = ([ATP] + 0.5 [ADP])/([ATP] + [ADP] + [AMP]). However, viability is aided if AEC can be maintained at a high value for as long as possible and so mechanisms are in place, particularly in skeletal muscle, to stabilize the AEC. The action of adenylate kinase (2 ADP ↔ ATP + AMP) allows some ATP to be resynthesized from ADP as it accumulates but this elevates [AMP]. However, if AMP is removed from the adenylate pool, the AEC ratio can be kept at a high value at the expense of an overall reduction in adenylate pool size. This mechanism is commonly seen in working skeletal muscle and also under a variety of environmental stress conditions that compromise energy reserves such as hypoxia, ischemia [14,15], freezing [13] or during torpor in hibernating mammals [16].

The removal of AMP from the adenylate pool is primarily the function of 5'-adenosine monophosphate deaminase (AMPD; EC 3.5.4.6) which hydrolyzes AMP to produce inosine monophosphate (IMP) and ammonium ion (NH_4 _^+^) [17]. AMPD action can lead to either a temporary drain of AMP (with IMP accumulating) or to a more permanent change if IMP is channeled into catabolic pathways leading ultimately to uric acid excretion. In skeletal muscle, the enzyme is well-known to be key to stabilizing the AEC during high-intensity muscle work in a range of vertebrate species [18-24]. The regulation of AMPD is complex and includes activation or inhibition by ions and metabolite effectors [25-27], reversible binding to myosin [18], oligomerization [28], and covalent modification [29]. Moreover, regulation by one of these factors can influence subsequent control by others. For example, binding of AMPD to myosin subunits alleviates allosteric inhibition of the enzyme from some sources, in addition to influencing substrate affinity and activity [26]. Phosphorylation of AMPD, in turn, may either influence or succeed binding, and has not yet been clearly elucidated [20].

The present study examines the regulation of AMPD in wood frog skeletal muscle. The enzyme from control (5°C acclimated) and frozen animals was compared to look for freeze-responsive changes in enzyme activity, kinetic parameters, phosphorylation state, temperature effects, and response to low molecular weight effectors.

## Results

### Activity and kinetic parameters of AMPD in crude extracts of skeletal muscle

AMPD is known to bind to myosin and a portion of total activity remains bound in the pellet after normal homogenization and centrifugation. The total activity of AMPD in wood frog muscle was 22–28 U/gww and the majority of activity was in the soluble fraction (Table 1). The percentage of AMPD in the bound form was 20.3 ± 0.13 % in control frogs and increased significantly to 35.4 ± 0.09 % in muscle from frozen frogs. Free AMPD showed a sigmoidal relationship between velocity and [AMP] with values for the Hill coefficient (n_H_) that were 1.76 ± 0.06 in muscle extracts from control frogs and 2.76 ± 0.10 in frozen frogs (Table 2). By contrast, bound AMPD displayed velocity versus substrate concentration profiles that were essentially hyperbolic (n_H _values of 1.03–1.12). Affinity of free AMPD for its substrate did not change between control and frozen states. However, *S*_0.5 _AMP of the bound enzyme was both significantly lower than the corresponding value for the free enzyme and changed between control and frozen states, *S*_0.5 _AMP of bound AMPD from muscle of frozen frogs being 3-fold higher than the value for control frogs.

**Table 1:** AMPD maximal activity (V_max_) and partitioning between free and bound forms in skeletal muscle from control (5°C-acclimated) and 24 h frozen frogs.

	V_max _(U/gww)	% of total activity
Control		
Free AMPD	17.6 ± 1.53	79.7 ± 6.46
Bound AMPD	4.5 ± 0.03	20.3 ± 0.13
Total	22.1 ± 1.56	100.0

Frozen		
Free AMPD	17.8 ± 0.69	64.6 ± 2.07 ^a^
Bound AMPD	9.8 ± 0.03 ^a^	35.4 ± 0.09 ^a^
Total	27.6 ± 0.72 ^a^	100.0

Data are expressed as units per gram wet weight (U/gww), means ± SEM, for determinations on muscle preparations from n = 8 individual frogs. ^a ^Significantly different from the corresponding value in control muscle by the Student's *t*-test, *P *< 0.05; percentage data were evaluated after arcsine √ transformation of the raw data.

**Table 2:** Kinetic parameters of AMPD in crude extracts of skeletal muscle from control (5°C-acclimated) and 24 h frozen wood frogs.

	*S*_0.5 _AMP (mM)	n_*H*_
Control		
Free AMPD	2.16 ± 0.11 ^b^	1.76 ± 0.06 ^bc^
Bound AMPD	0.46 ± 0.03 ^ac^	1.03 ± 0.07 ^ac^
Frozen		
Free AMPD	2.19 ± 0.15 ^b^	2.76 ± 0.10 ^ab^
Bound AMPD	1.50 ± 0.19 ^abc^	1.12 ± 0.09 ^a^

Data are means ± SEM, for determinations on muscle preparations from n = 4 individual frogs *S*_0.5 _is the substrate concentration that produces half-maximal enzyme velocity; n_*H *_is the Hill coefficient. Significantly different from ^a ^the corresponding value for free AMPD from control frogs, ^b ^the corresponding value for bound AMPD from control frogs, or ^c ^the corresponding value for free AMPD from frozen frogs using the Student's *t *test, *P *< 0.05.

### Endogenous effectors of AMPD

Soluble fractions of crude muscle extracts were centrifuged through small columns of Sephadex G-50 to remove ions and low molecular weight metabolites present in the crude extract. Desalting reduced *S*_0.5 _values for AMP by 23–35%, but values for the enzyme from control and frozen frogs remained similar (Table 3). The n_H _value for AMPD from control frogs also increased significantly but was similar to the value for frozen frogs.

**Table 3:** Kinetic parameters for free AMPD before and after removal of low molecular weight ions and metabolites by centrifugation through Sephadex G50 columns

	Before spun columns		After spun columns	
	*S*_0.5 _AMP (mM)	*n*_*H*_	*S*_0.5 _AMP (mM)	*n*_*H*_
	
Control	2.16 ± 0.11	1.76 ± 0.06	1.35 ± 0.08^a^	2.37 ± 0.17 ^a^
Frozen	2.19 ± 0.15	2.76 ± 0.10 ^a^	1.68 ± 0.21 ^a^	2.76 ± 0.27

Data are means ± SEM, for determinations on muscle preparations from n = 4 individual frogs. ^a ^– Significantly different from the corresponding free control value prior to desalting by the Student's *t*-test, *P *< 0.05.

### Allosteric effectors of AMPD

Sephadex G-50 filtered AMPD was assayed in the presence of potential effectors, to determine if these would affect activity. ATP only activated the free (soluble) form of AMPD, whereas ADP activated both free and bound enzyme forms (Table 4). The *K*_*a *_ATP decreased significantly by 69% for AMPD from muscle of frozen frogs, compared with the value for control frogs. ATP activated AMPD from control frogs by 1.53-fold, whereas in frozen frogs, the fold activation was 2.69. Similarly, the *K*_*a *_ADP for both free and bound forms of AMPD was lower than the corresponding values for the enzyme from control frogs. The *K*_*a *_ADP for bound AMPD in control frogs was significantly higher than the corresponding free value for the enzyme from either control or frozen frogs. ADP activated free AMPD in control frogs by 2.17-fold, whereas in frozen frogs, this increased to 5-fold. A similar increase was noted for the bound forms of AMPD. ADP activated bound AMPD in control frogs by 1.25-fold and this increased to 3.42-fold in frozen frogs.

**Table 4:** Adenylate activation of wood frog skeletal muscle AMPD from control versus frozen frogs

	Control		Frozen	
	
	Free AMPD	Bound AMPD	Free AMPD	Bound AMPD
*K*_*a *_Mg·ATP, mM	1.90 ± 0.03	NE	0.63 ± 0.04 ^a^	NE
Mg·ATP fold-activation	1.53 ± 0.09	NE	2.69 ± 0.20 ^a^	NE
*K*_*a *_Mg·ADP, mM	1.12 ± 0.15 ^b^	3.75 ± 0.37	0.54 ± 0.02 ^ab^	1.14 ± 0.10 ^a^
Mg·ADP fold-activation	2.17 ± 0.29	1.25 ± 0.21	5.01 ± 0.69 ^a^	3.42 ± 0.24 ^a^

Data are means ± SEM, for determinations on Sephadex G50-filtered muscle extracts from n = 4 individual frogs. Significantly different from ^a ^the corresponding value in control frogs, ^b ^the corresponding value for bound AMPD in control frogs, by the Student's *t*-test, *P *< 0.05. NE, no effect. *K*_*a *_is the activator concentration that produces half-maximal activation; values were determined at 0.5 mM AMP. *K*_*a *_and fold activation values were calculated using a nonlinear least-squares regression kinetics program [39].

GTP was a very strong inhibitor with *I*_50 _values of just 0.08–0.15 mM (Table 5). *I*_50 _GTP of free AMPD was about 50% lower for the enzyme from frozen frogs but the value for bound AMPD was unaffected by the transition to the frozen state. IMP is the product of the AMPD reaction. IMP inhibited bound AMPD from muscle of frozen frogs with an *I*_50 _of 0.23 ± 0.02 mM but interestingly, did not inhibit the enzyme in any other situation at the IMP concentrations tested (up to 10 mM). Salts also inhibited AMPD; all three salts tested had Cl^- ^as the anion so differences in inhibitory effects are due to cation effects. Na^+ ^and K^+ ^were very weak inhibitors of free AMPD with *I*_50 _values in the range of 650–970 mM but both cations had much stronger effects on bound AMPD with *I*_50 _values between 130–160 mM. NH_4 _^+^, another product of the AMPD reaction, inhibited the free enzyme with *I*_50 _values in the 250–275 mM range. The effects of NH_4 _^+ ^on the bound enzyme could not be tested because NH_4 _^+ ^is the product being measured by the GDH-coupled assay used for the bound enzyme.

**Table 5:** Inhibitor effects (*I*_50 _values) on AMPD from skeletal muscle of control versus frozen wood frogs

	Control		Frozen	
	
	Free AMPD	Bound AMPD	Free AMPD	Bound AMPD
Mg·GTP, mM	0.15 ± 0.02	0.08 ± 0.01	0.08 ± 0.01 ^a^	0.10 ± 0.01
IMP, mM	NE	NE	NE	0.23 ± 0.02 ^a^
KCl, mM	660 ± 44	148 ± 34	702 ± 21 ^a^	162 ± 24
NaCl, mM	973 ± 20	141 ± 31	824 ± 26 ^a^	133 ± 21
NH_4_Cl, mM	276 ± 5.0	ND	254 ± 10	ND

Data are means ± SEM, for determinations on Sephadex G50-filtered muscle extracts from n = 4 individual frogs. *I*_50 _is the inhibitor concentration that reduces enzyme velocity by 50%. ^a ^– Significantly different from the corresponding value for muscle from control frogs as assessed by the Student's *t*-test, *P *< 0.05. NE, no effect. ND, not determined. *I*_50 _values were measured at an AMP concentration of 0.5 mM.

### Temperature and cryoprotectant effects

AMPD activity was assayed in the presence versus absence of glucose, the cryoprotectant that is present in high concentrations in tissues of frozen frogs. Activity was also assessed at high (25°C) and low (5°C) temperatures. Addition of high glucose (250 mM) significantly decreased the *S*_0.5 _AMP for the enzyme from both control and frozen frogs when assayed at high temperature, the effect being particularly strong (84% reduction) for the enzyme from frozen frogs (Table 6). In contrast, *S*_0.5 _AMP increased significantly when the enzyme was assayed at a cold temperature (5°C), close to the freezing temperatures when frogs would naturally experience high glucose. The combined effects of cold temperature and high glucose together resulted in 1.9- and 1.4-fold increases in *S*_0.5 _AMP for the enzyme from control and frozen frogs, respectively. The *S*_0.5 _AMP for AMPD from frozen frogs under these conditions was still markedly higher (by 2.5-fold) than the corresponding value for the enzyme from control frogs.

**Table 6:** Effects of assay temperature and glucose on the *S*_0.5 _AMP for bound AMPD from skeletal muscle of control versus frozen wood frogs.

	*S*_0.5 _AMP (mM)	
	
	Control	Frozen
25°C, no glucose	0.46 ± 0.03	1.50 ± 0.19
25°C, 250 mM glucose	0.30 ± 0.04 ^a^	0.24 ± 0.03 ^a^
5°C, no glucose	0.70 ± 0.03 ^a^	1.77 ± 0.03 ^a^
5°C, 250 mM glucose	0.87 ± 0.04 ^a^	2.14 ± 0.04 ^a^

Data are means ± SEM, for determinations on Sephadex G50-filtered muscle extracts from n = 4 individual frogs. ^a ^– Significantly different from the corresponding value at 25°C without glucose as assessed by the Student's *t*-test, *P *< 0.05.

### Reversible phosphorylation

**Figure 1:**
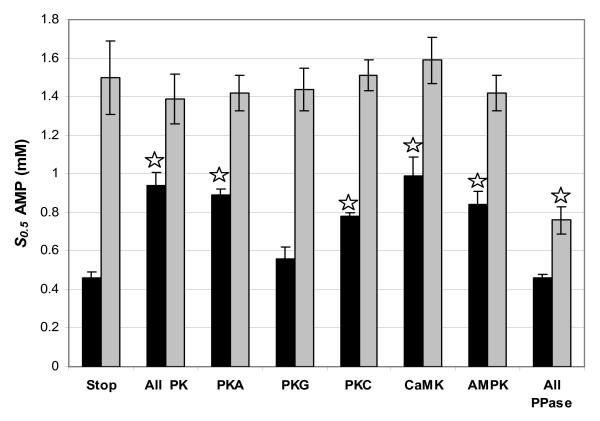
**Effect of reversible phosphorylation on the substrate affinity for AMP of bound AMPD from skeletal muscle of control (black bars) and frozen (gray bars) wood frogs**. Data are means ± SEM, n = 4 determinations on separate preparations of enzyme. Stars indicate kinetic parameters that are significantly different from the corresponding value for incubations in stop buffer as assessed by the Student's *t *test, *P *< 0.05.

Many enzymes are targets of covalent modification by protein kinases and protein phosphatases. To determine whether reversible phosphorylation affected bound AMPD, incubations were set up in which enzyme phosphorylation or dephosphorylation was promoted by stimulating the activities of endogenous protein kinases and phosphatases. Incubations that stimulated all protein kinases resulted in a significant 2-fold increase in *S*_0.5 _AMP of bound AMPD from muscle of control frogs (Figure 1). Similarly, *S*_0.5 _AMP of control AMPD increased significantly when the activities of PKA, PKC, CaMK, or AMPK (but not PKG) were stimulated individually. However, stimulation of these protein kinases had no effect on the substrate affinity of AMPD from muscle of frozen frogs.

When incubations stimulated protein phosphatase activities instead the opposite effect was seen. The *S*_0.5 _AMP of muscle AMPD from frozen frogs decreased significantly by 49 % after stimulation of phosphatase activities. Conversely, stimulation of protein phosphatases had no noticeable effect on *S*_0.5 _AMP of the enzyme from control frogs.

## Discussion

Freezing imposes long term ischemia upon wood frog organs and while frozen, organ ATP levels decline and so can the AEC [13]. Freeze-thaw cycles accelerate the decline in AEC, and it can be expected that several freeze-thaw cycles must be endured over a typical winter [30]. Studies of wood frog liver and leg muscle showed decreasing [ATP] in both organs during freezing but whereas a noticeable drop in AEC occurred in liver, no appreciable change was noted in leg muscle over several freeze-thaw cycles. This implies that skeletal muscle has a metabolic strategy in place to support AEC at high values for as long as possible [30]. This strategy appears to involve the action of AMPD in draining AMP from the muscle adenylate pool and hence, the present study addressed the regulation of skeletal muscle AMPD, comparing enzyme properties from unfrozen versus frozen wood frogs.

An initial analysis of the maximum activity (*V*_*max*_) and apparent substrate affinity (*S*_0.5_) of wood frog AMPD in soluble extracts of skeletal muscle found no change in these enzyme parameters between control and frozen states (Tables 1 and 2). Removal of endogenous low molecular weight effectors using Sephadex G50 spun columns reduced the *S*_0.5 _for AMP of the free enzyme from both control and frozen muscle by similar amounts and altered the *n*_*H *_value for control AMPD (Table 3). This indicates that one or more endogenous inhibitors (e.g. GTP) are present in crude extracts. Further analysis of the responses by free AMPD to activators and inhibitors showed differences between the enzyme from control and frozen states (Tables 4 and 5). The enzyme from muscle of frozen frogs had significantly lower *K*_*a *_values for ATP and ADP and the *I*_50 _GTP was also reduced by about 50%. Overall, then free AMPD in muscle of frozen frogs was more sensitive to high energy phosphates than in the control state. Given that the levels of these high energy phosphates also fall *in vivo *in muscle during freezing, these changes in the properties of the enzyme could possibly maintain the relative sensitivity of the enzyme to regulatory control by high energy phosphates in the frozen state.

A comparison of bound AMPD from muscle of control versus frozen frogs showed that control AMPD had much higher affinity for AMP than did the enzyme from frozen muscle (Table 2). However, bound AMPD from frozen frogs had a lower *K*_*a *_ADP (just 30% of the value for control frogs) which would increase bound AMPD sensitivity to the product of ATP hydrolysis in frozen muscle and potentially link it more closely to muscle energetics. Interestingly, IMP exclusively inhibited the bound form of AMPD from frozen frogs, but did not inhibit free forms of the enzyme, or bound AMPD in control frogs. The unique inhibitory effect of IMP on bound AMPD from frozen frogs suggests that this form of the enzyme exists in a distinct state, where it can be tightly controlled by key metabolites (adenylates, GTP, IMP) that reflect the energy status of skeletal muscle while frozen.

Binding to myosin is a major regulatory feature of AMPD that has been extensively explored [18-21,26,31]. The amount of AMPD that was bound in wood frog muscle increased from 20% in control frogs to 35% in frozen animals suggesting that a greater association of AMPD with myofibrils benefited metabolism in frozen muscle. This response may be triggered by the anoxic/ischemic conditions that accrue as freezing progresses. Low oxygen stress is known to enhance AMPD binding in muscle from other species [14]. Indeed, Rundell et al. [31] reported that muscle AMPD remained bound as long as ischemic conditions persisted. Bound AMPD had a greater affinity for AMP substrate than free AMPD in both control and frozen frogs and bound AMPD also showed hyperbolic AMP kinetics compared with the sigmoidal relationship seen for free AMPD. Bound AMPD was less sensitive to ADP activation than was its comparable free form but was much more strongly inhibited by Na^+ ^and K^+ ^ions in both control and frozen frogs.

Not only is AMPD distributed between free and bound states but myofibril-bound AMPD is also subject to covalent modification by protein kinases and phosphatases [20]. Indeed, cardiac AMPD was phosphorylated by at least one major protein kinase, PKC [29]. Protein kinases and phosphatases, although often soluble, can be compartmentally restricted through anchoring and scaffolding proteins, allowing them access to phosphorylate and dephosphorylate bound enzymes such as AMPD [32,32]. To determine whether wood frog muscle AMPD was subject to reversible phosphorylation, preparations of bound AMPD from control frogs were incubated under conditions that stimulated the action of endogenous protein kinases (PKA, PKC, CaMK, or AMPK). After protein kinase treatment, the enzyme consistently showed reduced affinity for AMP (*S*_0.5 _increased by about 2-fold) (Fig. 1). Conversely, conditions that promoted the dephosphorylation of bound AMPD from frozen frogs caused an increase in AMP affinity (*S*_0.5 _decreased by about half). This implies that wood frog muscle AMPD is regulated by reversible phosphorylation when bound, and that in the transition from the control to frozen states, the phosphorylation state is modified. The data are consistent with a low phosphate form of bound AMPD in control frogs and a high phosphate form in frozen frogs and with one of the actions of phosphorylation being to reduce the affinity of the bound enzyme for AMP. Enhanced phosphorylation of bound AMPD may also be responsible for other changes in the kinetic properties of the bound enzyme from frozen muscle such as the change in sensitivity to ADP activation, the unique inhibition by IMP, and the response of the enzyme to high glucose.

Freezing alters cytosolic conditions in frog cells, in particular by greatly elevating the concentrations of glucose which acts as a cryoprotectant. Hence, effects of high glucose on enzymes need to be considered as well as potential interactions between cryoprotectants and temperature. High concentrations of glucose increased the substrate affinity for AMP of bound AMPD when the enzyme was assayed at 25°C but had relatively little effect on *S*_0.5 _at low assay temperatures. By contrast, low temperature assay at 5°C decreased AMP substrate affinity of the enzyme from both control and frozen frogs. Overall, the additive effects of high glucose and low temperatures led to a substantial reduction in AMPD affinity for its substrate compared with the situation at high temperature without glucose with increases in *S*_0.5 _AMP of 2-fold for the bound enzyme from control frogs and ~25% for AMPD from frozen frogs. Interestingly, both of these factors coupled together led to a combined decrease in substrate affinity of AMPD. Hence, both temperature change and high glucose could influence the function of AMPD between unfrozen and frozen states.

Many previous studies have focused on AMPD in working skeletal muscle and found that the enzyme is more active and more bound under exercising conditions. By contrast, the present study suggests that in the frozen state, bound AMPD is less active with reduced substrate affinity. This agrees with a study on AMPD in muscle of hibernating mammals that found that substrate affinity was reduced at low temperatures in the torpid state [16]. AMPD was also inhibited in rabbit heart experiencing hypoxia [34]. In that study, AMP deamination was high early in the ischemic period, and this served to preserve the AEC. After prolonged ischemia, however, AMPD was stably inhibited and IMP accumulation was implicated in AMPD inhibition. Hence, it can be recognized that AMPD is involved in two forms of energy stress in muscles – that caused by burst muscle exercise (e.g. active frogs jumping) and that caused by ischemia/hypoxia due to oxygen limitation. In both cases, an initial decrease in ATP and increase in ADP and AMP leads to an increase in the amount of bound AMPD localized physically with the subcellular area(s) where AMP is accumulating. However, whereas energy stress due to exercise is typically short-lived until fatigue sets in and then quickly reversed, energy stress due to hypoxia/ischemia can be of indefinite term (e.g. days or weeks of freezing for wood frogs). Therefore, it appears that species that are tolerant of freezing (or of anoxia or of deep torpor) allow their total adenylate pool to be partially depleted as an early response to stress (using AMPD to drain the pool) but then need to arrest the process and re-establish homeostasis in a hypometabolic state that can be sustained over the long term. Phosphorylation of bound AMPD may be the key in this phase. Bound AMPD in 24 h frozen frogs is a high phosphate form with relatively low affinity for AMP and strongly inhibited by IMP. Hence, it is likely that enzyme activity *in vivo *is low over the long term in frozen muscle. Effects of low temperature and high glucose (both conditions in frozen muscle) on *S*_0.5 _AMP support the same conclusion. Numerous studies with multiple animal systems of diverse phylogenetic origins have shown that one of the key mechanisms that regulates animal transitions into hypometabolic states is protein phosphorylation [11]. The present data suggest that AMPD activity is similarly integrated into the needs of the hypometabolic, frozen state by phosphorylation of the bound enzyme.

Reversal of these processes during thawing, including enzyme dephosphorylation, temperature rise and glucose decrease, will all contribute to a return by AMPD to normal function, as in control muscle. Recovery of other enzymatic systems will also allow a recovery of muscle high energy reserves (e.g. total adenylate and creatine phosphate pools), the clearance of glucose and glycolytic end products, the re-establishment of aerobic metabolism, and the reactivation of a variety of energy-expensive cell functions that are typically strongly suppressed in hypometabolic states including the activities of ion motive ATPases (needed for muscle contractile activity), transcription and translation [11,35]. Given the variety of metabolic functions that must recover (or be repaired) after thawing, it is perhaps not surprising that hind leg reflex contractions took more than 4 hours to return in frogs thawed at 6–8°C [36] and that isolated wood frog skeletal muscles still showed marked impairment of locomotor endurance up to 96 h post-freeze [37].

## Conclusion

Overall, this study shows that skeletal muscle AMPD from control and frozen frogs has significantly different kinetic parameters that would alter enzyme function during freezing. Binding, allosteric effectors, glucose, temperature and reversible phosphorylation all play roles in regulating wood frog AMPD and adjusting enzyme function for optimal action in the regulation of cellular adenylate levels in ischemic frozen muscle. For example, phosphorylation of bound AMPD may be responsible for some of the unique properties of the bound enzyme in frozen muscle such as the change in sensitivity to ADP activation, inhibition by IMP, and the response of the enzyme to high glucose. Reversible phosphorylation control of AMPD could also have a critical role in coordinating AMPD function with respect to other enzymes of muscle energy metabolism to minimize overall energy use while frozen and achieve maximum extension of viability.

## Methods

### Animals and chemicals

Male wood frogs were captured from spring breeding ponds in the Ottawa area. Animals were washed in a tetracycline bath, and placed in plastic containers with damp sphagnum moss at 5°C for two weeks prior to experimentation. Control frogs were sampled directly from this condition. For freezing exposure, frogs were placed in closed plastic boxes with damp paper toweling on the bottom, and put in an incubator set at -3°C. A 45 min cooling period was allowed during which the body temperature of the frogs cools to below -0.5°C (the equilibrium freezing point of wood frog body fluids) and nucleation is triggered due to skin contact with ice crystals formed on the paper toweling [38]. Subsequently, timing of a 24 h freeze exposure began. Both control and experimental frogs were sacrificed by pithing, followed by rapid dissection, and freezing of tissue samples in liquid nitrogen. Frozen tissues were stored at -80°C until use. Conditions for animal care, experimentation, and euthanasia were approved by the Carleton University Animal Care Committee in accordance with guidelines set down by the Canadian Council on Animal Care. Biochemicals and coupling enzymes were purchased from Sigma Chemical Co. (St. Louis, MO) or Boehringer-Mannheim (Montreal, PQ).

### Preparation of tissue extracts

Samples of frozen skeletal muscle were homogenized 1:5 w:v in ice-cold buffer A (50 mM MOPS, 2 mM EDTA, 2 mM EGTA, 50 mM β-glycerophosphate, 10 mM β-mercaptoethanol, pH 7.0), with a few crystals of the protease inhibitor, phenylmethylsufonyl fluoride, added immediately prior to homogenization with a Polytron homogenizer at 50% of full power. Homogenates were centrifuged in a Hermle Z 360 K centrifuge for 30 min at 10,000 × *g *and the supernatant was removed.

In cases where ion or metabolite effects on AMPD were being investigated, crude extracts were then centrifuged through small columns of Sephadex G-50 to remove endogenous enzyme effectors. To do this, 5 ml syringe barrels were plugged with glass wool and packed with Sephadex G-50 equilibrated in buffer A. Packed syringes were centrifuged in a bench-top IEC clinical centrifuge at full power for 1 min and eluant was discarded. A 500 μl aliquot of supernatant was then layered onto the column followed by a second centrifugation as above. The eluant was collected and stored on ice.

For experiments where bound AMPD activity in the pellet was assessed, the pellet from the original homogenate was resuspended in a volume of buffer A equal to that used for the original homogenization. The resuspension was then centrifuged at 10,000 × *g *for 30 min and the supernatant was decanted. The procedure was repeated twice more and then the final pellet was resuspended in homogenization buffer, and used for assay.

### Enzyme assay

Enzyme activity was assayed in a 200 μl final volume using a Thermo Labsystems Multiskan Spectrum microplate reader. Standard assays were conducted at 25°C. Optimal assay conditions for AMPD were 50 mM MOPS buffer (pH 7.2), 5.0 mM AMP, and 20 μl of enzyme extract, monitored at 285 nm. One unit of enzyme activity is defined as the amount that produced 1 μmol of IMP per minute at 25°C. For assays of bound AMPD, in which a resuspension of the pellet was assayed, a glutamate dehydrogenase (GDH)-coupled assay was used. Optimal conditions were 50 mM MOPS buffer (pH 7.2), 5.0 mM AMP, 0.15 mM NADH, 7.5 mM α-ketoglutarate, 1 unit of GDH and 10 μl of resuspended pellet, monitored at 340 nm. One unit of enzyme activity is defined as the amount that consumed 1 μmol of NADH per minute at 25°C.

### In vitro incubations to stimulate endogenous kinases and phosphatases

To assess the effect of reversible phosphorylation on the bound form of AMPD, incubations were prepared under conditions that stimulated the activities of endogenous protein kinases or protein phosphatases and the resulting effects on AMPD were assayed. Pellets containing bound AMPD were prepared and washed as above and then resuspended in 50 mM MOPS, pH 7.2, and 10 mM β-mercaptoethanol, aliquoted into different tubes, and incubated under the following conditions, in a final volume of 1 mL:

*(A) "Stop" conditions: *50 mM β-glycerophosphate, 2 mM EDTA and 2 mM EGTA to inhibit both protein kinase and phosphatase activities.

*(B) Stimulation of endogenous protein kinases: *5 mM Mg·ATP, 50 mM β-glycerophosphate and either (1) 1 mM cAMP to stimulate PKA; (2) 1 mM cGMP to stimulate PKG; (3) 1.3 mM CaCl_2 _+ 7 μg/mL phorbol myristate acetate to stimulate PKC; (4) 1 mM AMP to stimulate AMPK; (5) 1 U of calmodulin + 1.3 mM CaCl_2 _to stimulate CaMK; (6) all of these components to stimulate all kinases together.

*(C) Stimulation of endogenous protein phosphatases: *5 mM MgCl_2 _and 5 mM CaCl_2 _to stimulate all protein phosphatases.

Samples were incubated for 4 hours at 4°C. Incubations were then centrifuged at 10,000 × *g *for 30 minutes, and the supernatant was discarded. The pellet was resuspended in 1 mL of buffer A, used for the original tissue homogenization. The resuspension was then centrifuged at 10,000 × *g *for 30 min and the supernatant was decanted. The procedure was repeated twice more (to ensure removal of all low molecular weight effectors) and then the pellet was resuspended in homogenization buffer, and used for assay.

### Kinetic parameters

Enzyme kinetic constants were determined using a nonlinear least-squares regression computer program [39]; substrate affinity data were fitted to Hill plots.

## Abbreviations

AEC: adenylate energy charge; AMPD: AMP deaminase; AMPK: AMP dependent protein kinase; CAMK: calcium/calmodulin dependent protein kinase; PKA: cyclic 3',5' adenosine monophosphate-dependent protein kinase; PKC: Ca^2+ ^and phospholipid dependent protein kinase; PKG: cyclic 3',5' guanosine monophosphate-dependent protein kinase.

## Authors' contributions

CAD carried out all the experimental procedures and was involved in drafting and revising the manuscript. KBS designed and supervised the study and was involved in drafting and revising the manuscript. Both authors read and approved the final manuscript.
